# Exploratory Prospective Assessment of Instrumental Skin Changes and Patient-Reported Outcomes After Microneedling of the Neck and Décolleté

**DOI:** 10.3390/jcm15145480

**Published:** 2026-07-13

**Authors:** Justyna Pająk-Majewska, Małgorzata Ponikowska, Danuta Nowicka

**Affiliations:** University Centre of General Dermatology and Oncodermatology, Faculty of Medicine, Wroclaw Medical University, 50-556 Wroclaw, Poland; jpajakowna@gmail.com (J.P.-M.); malgorzata.ponikowska@umw.edu.pl (M.P.)

**Keywords:** microneedling, skin hydration, skin firmness

## Abstract

**Objectives**: Microneedling is a minimally invasive procedure intended to improve skin quality through controlled mechanical stimulation of the dermis; however, objective data on its effects remain limited. This study aimed to evaluate regional changes in skin hydration and firmness after a series of microneedling treatments of the neck and décolleté using corneometry, indentometry, and patient-reported outcomes. **Methods**: The study included 30 female patients who underwent three microneedling sessions performed at 4-week intervals. Instrumental measurements (corneometry and indentometry) and photographic documentation were obtained before treatment initiation and 4 weeks after completion of the treatment series. Statistical analysis included linear mixed models (LMMs), Spearman’s rank correlation, and Friedman’s test for repeated measurements. **Results**: Microneedling produced heterogeneous responses depending on anatomical location. In corneometry analyses excluding the control point, a significant increase in hydration was observed only at point 4, while no significant changes were detected at the remaining treatment sites. Similarly, indentometry demonstrated a localized improvement in firmness exclusively at point 4, reflected by decreased indentation values after treatment. In models including the control point, no generalized time effect was observed for either corneometry or indentometry; however, significant effects of anatomical location and location-by-time interactions persisted, indicating site-dependent variability in treatment response. Age was not associated with global treatment outcomes; however, a significant age-by-location interaction was identified for indentometry. No significant correlations were found between objective instrumental measurements and subjective assessments of hydration or firmness, despite most participants reporting perceived clinical improvement. Additionally, the depth at which pinpoint bleeding occurred increased significantly across subsequent treatment sessions. **Conclusions**: Microneedling of the neck and décolleté resulted in localized rather than generalized instrumental skin changes, with treatment response strongly influenced by anatomical location. Objective measurements did not correlate with patient-reported improvement, highlighting the complexity of assessing esthetic treatment outcomes. However, the results should be treated cautiously due to the exploratory nature of this study.

## 1. Introduction

Microneedling is a widely used minimally invasive procedure aimed at improving skin quality through controlled mechanical stimulation of the dermis [1,2,3,4,5,6,7]. The technique has gained substantial popularity in esthetic dermatology because of its favorable safety profile, relatively short recovery time, and broad range of clinical applications, including skin rejuvenation, scar remodeling, and wrinkle reduction [2,3,4,5,6,7]. Compared with classic needle mesotherapy, microneedling is often better tolerated by patients due to the use of multiple fine needles and controlled penetration depth [8].

The procedure is frequently combined with topical active substances to enhance transdermal delivery [3,4,5]. Treatment depth is usually adjusted according to anatomical location and therapeutic indication, with the endpoint commonly defined by the appearance of fine pinpoint bleeding [1,2,3,4,7]. In the neck and décolleté region, penetration depths of approximately 1.5–3 mm are most commonly used [3]. Despite the growing popularity of microneedling in esthetic practice, objective instrumental evaluation of treatment outcomes in these anatomical areas remains limited.

Most published studies assessing microneedling efficacy have focused primarily on subjective clinical improvement, photographic evaluation, or facial rejuvenation [9,10,11,12,13,14,15,16,17]. Data regarding the neck and décolleté are scarce, and studies using objective bioengineering methods such as corneometry or indentometry are particularly limited. In the context of this study, objective instrumental evaluation refers to device-based, quantitative assessment of selected skin parameters, performed independently of the patient’s subjective perception. Corneometry was selected to assess stratum corneum hydration, whereas indentometry was used to evaluate skin firmness and mechanical resistance to deformation. These methods were chosen to complement patient-reported outcomes and to provide measurable indicators of treatment-related changes in skin hydration and firmness. Moreover, little is known about the relationship between objectively measured skin changes and patient-reported treatment outcomes.

Therefore, the aim of the present study was to evaluate regional changes in skin hydration and firmness after a series of three microneedling treatments of the neck and décolleté using corneometry, indentometry, and subjective patient assessments. Additionally, the study investigated whether treatment response was influenced by age and anatomical location, as well as whether objective instrumental findings correlated with perceived clinical improvement.

## 2. Materials and Methods

This was a prospective study conducted from January 2024 to November 2025. The study included 30 female patients aged 28–50 years (mean 37.2 ± 7.2 years) with Fitzpatrick skin phototypes I–III. Décolleté photoaging severity was assessed at baseline using the Glogau scale (grades 1–3).

Exclusion criteria included allergy to study-related materials, dermal filler treatment within the previous 12 months, tendency toward keloid formation, active herpes simplex infection, autoimmune diseases, coagulation disorders, active inflammation or infection, and any medication or condition that could potentially influence the study outcomes.

All participants underwent a series of three microneedling treatments performed at 4-week intervals (±1 week). Instrumental measurements and photographic documentation were obtained at baseline and 4 weeks after completion of the treatment series.

Microneedling treatments were performed using a Dermapen 4 device equipped with 16 sterile single-use needles (DermapenWorld Pty Ltd., Belrose, Sydney, NSW, Australia). Before each procedure, the skin was cleansed and disinfected. No mesotherapy formulations, platelet-rich plasma, growth factors, or other active topical products were applied before or during treatment.

Sterile 0.9% sodium chloride solution was used as the sole glide medium throughout the procedure. The device was operated at the maximum speed setting. Four passes were performed over the entire treatment area in four directions: vertical, horizontal, diagonal from left to right, and diagonal from right to left.

Needle penetration depth was adjusted individually for each anatomical area and participant according to skin thickness and the observed tissue response. The clinical endpoint was the appearance of pinpoint bleeding. Consequently, both the needle depth and total treatment duration varied between participants and anatomical areas. Treatment was continued until the predefined clinical endpoint had been achieved throughout the treated area.

All procedures were performed by the same physician to ensure procedural consistency. No topical anesthesia was used because local anesthetics such as lidocaine or mepivacaine may interfere with normal tissue repair processes [1,18].

Objective skin assessment was performed using Courage + Khazaka devices (Courage + Khazaka electronic GmbH, Cologne, Germany), including a corneometer (System Multi Probe Adapter MP) and an IDM 800 indentometer. Corneometry was used to assess stratum corneum hydration based on the dielectric properties of the skin [19,20]. Indentometry measured skin deformation under a fixed applied force, with lower indentation values corresponding to greater tissue firmness [21,22,23].

**Figure 1:**
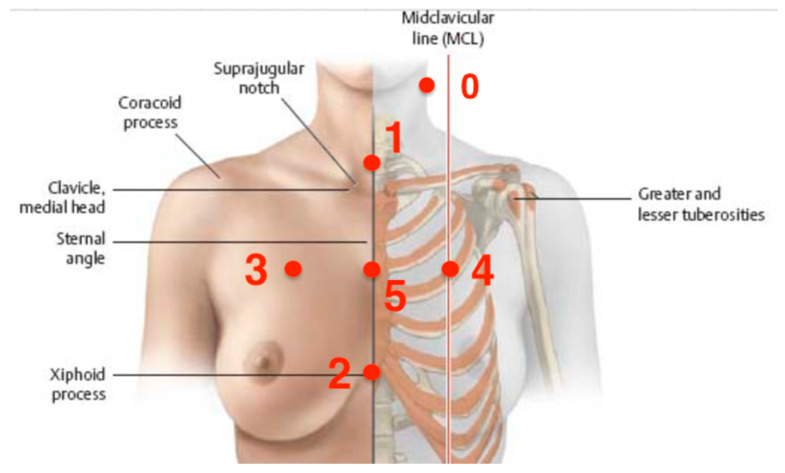
Locations of measurement points 0–5; point 0 represents the untreated control area.

Measurements were obtained at six predefined anatomical points (0–5) within the neck and décolleté region (Figure 1). Measurement points were defined as follows: point 0, upper neck/supraclavicular region; point 1, upper chest/neck transition region near the sternoclavicular area; point 2, lower medial décolleté region; point 3, right anterior chest/décolleté region along the midclavicular line; point 4, left anterior chest/décolleté region along the midclavicular line; and point 5, central décolleté region adjacent to the sternum. Points 3 and 4 represented anatomically comparable right- and left-sided anterior chest regions. Points 1–5 underwent microneedling treatment, whereas point 0 served as an adjacent untreated control area. The measurement points were selected a priori to provide spatial sampling across clinically distinct subregions of the neck and décolleté, including central and lateral areas and different cranio-caudal levels. This distribution was intended to account for potential regional variation in skin properties related to anatomical location, underlying tissue, mobility, and exposure to environmental factors. The points were not selected to represent individual dermatomes. Point 0 was located in an adjacent untreated area and was included as an internal control to estimate temporal variability in untreated skin, whereas points 1–5 represented treated areas within the neck and décolleté. Because the control point was adjacent rather than anatomically identical to all treated sites, comparisons involving point 0 were interpreted as supportive rather than as a fully matched site-specific control. The study was designed as a within-subject comparison, in which treated and untreated control points were assessed in the same participant. This approach was chosen to reduce interindividual variability in baseline skin characteristics, which may substantially differ between participants and could otherwise confound the assessment of treatment-related changes.

Corneometer and indentometer measurements were performed before and after the treatment series at all measurement sites. Measurements were always taken after the equipment had been calibrated, in a room with constant temperature and humidity. Three measurements were taken at each location, and the average was used in the calculations. The instrument measures the penetration depth of the pin in 3 mm, the shape of the indenter has been deliberately chosen to be cylindrical and not roundish in order to allow an absolute constant contact area. Additionally, the needle depth at which pinpoint bleeding occurred during each session was recorded.

Patient-reported outcomes included subjective assessments of skin hydration and firmness obtained before and after treatment using two separate single-item 10-point Likert-type scales. For each parameter, a score of 0 indicated the worst perceived condition and a score of 10 indicated the best perceived condition, with higher scores reflecting better perceived skin hydration or firmness.

Statistical analysis was performed using linear mixed models (LMMs) to evaluate the effects of time, anatomical location, and age on instrumental measurements. Separate analyses were conducted for models including and excluding the untreated control point. Correlations between objective measurements and subjective assessments were analyzed using Spearman’s rank correlation coefficient. Changes in needle depth associated with pinpoint bleeding across subsequent sessions were evaluated using Friedman’s test for repeated measurements, followed by Nemenyi post hoc pairwise comparisons to account for multiple comparisons. All statistical tests were two-sided, and *p*-values below 0.05 were considered statistically significant.

## 3. Results

Primary analyses focused on treated points 1–5. Additional analyses, including point 0 were performed to compare changes at treated sites with variability observed in untreated skin. Point 0 served as an untreated control area and was measured at the same time points as the treated sites, before and after the treatment series.

**Figure 2:**
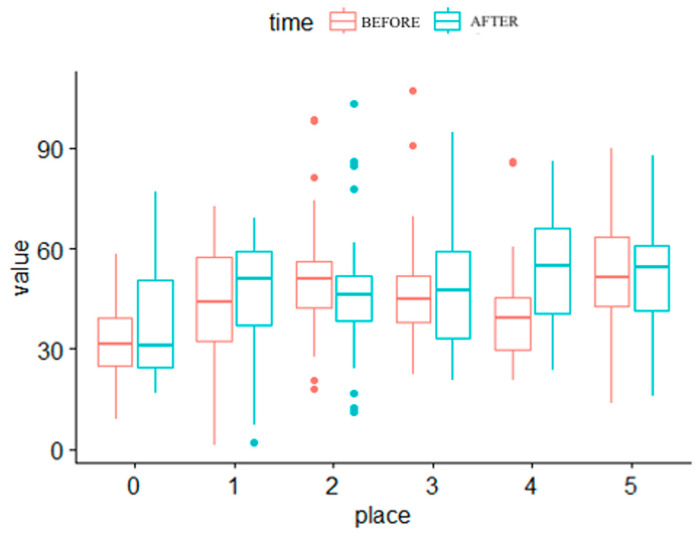
Corneometer results according to location and time. An increase in values was observed at all points except point 2.

Corneometer values did not show a significant overall increase after treatment (Table 1, Appendix A Table A1); however, corneometer values increased numerically at all points except point 2 (Figure 2). Baseline hydration differed among anatomical sites, and the magnitude of change varied significantly according to anatomical location, as demonstrated by the significant time-by-location interaction in both models. When only treated sites were analyzed, point 4 exhibited a significantly greater increase in corneometer values than the reference treatment site (point 1). In the age-adjusted model, age was not significantly associated with corneometry outcomes, and no significant age-by-time interaction was observed. The effects of anatomical location and time-by-location interaction remained significant after adjustment for age, indicating that regional variability, rather than age, was the main factor associated with corneometry results (Table 2, Appendix A Table A2).

Indentometry showed heterogeneous, location-dependent variability in skin firmness measurements (Table 3, Appendix A Table A3). In the LMM, including the untreated control point, no significant overall effect of time was observed, indicating no uniform change in indentometer readings after the treatment series. However, anatomical location and the time-by-location interaction were significant, suggesting that indentometer values differed between measurement sites and that changes over time varied by anatomical location. When the analysis was restricted to treated points only, anatomical location remained significant, whereas the overall effect of time and time-by-location interaction were not significant. In the age-adjusted model including point 0, age was not significantly associated with indentometry outcomes, and no significant age-by-time interaction was observed (Table 4, Appendix A Table A4). However, a significant age-by-location interaction was identified, indicating that the relationship between age and indentometer readings differed across anatomical sites.

**Figure 3:**
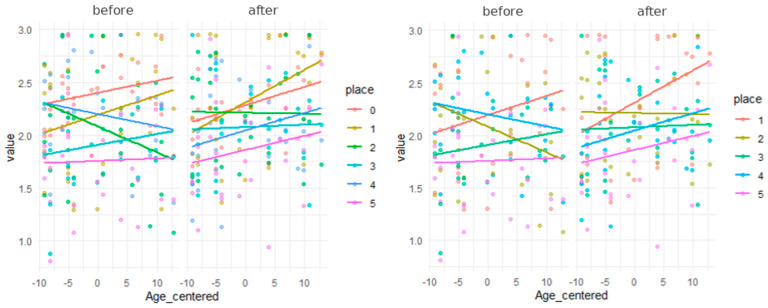
Age and change in indentometer readings, including point 0 (**left panel**) and excluding point 0 (**right panel**).

Age was not significantly associated with overall corneometry or indentometry outcomes (Table 2 and Table 4). No significant age-by-location interaction was observed for corneometry (Table 2). For indentometry, however, a significant overall age-by-location interaction was identified, indicating that the association between age and indentometer readings differed across anatomical locations (Table 4). In the parameter estimates, this interaction was significant for point 2 compared with the reference location (Figure 3).

Patient-reported assessments showed a limited link with instrumental measurements. In the model evaluating subjective hydration and corneometry, the patient-reported hydration score was not significantly associated with corneometer readings, and no significant interactions with anatomical location or time were identified. Although a significant time effect was observed for corneometry, the absence of a significant association between subjective hydration scores and corneometer values indicates that patient-reported hydration did not align with objective instrumental measurements. Similarly, in the model evaluating subjective firmness and indentometry, the patient-reported firmness score was not significantly associated with indentometer readings, and no significant interactions with anatomical location or time were observed. Anatomical location remained significantly associated with indentometer values, indicating site-dependent variability in mechanical skin measurements. Results for the relationships between instrumental results and patients’ subjective assessments are shown in Table 5 and Appendix A Table A5.

Despite the limited changes detected instrumentally, most participants reported subjective improvement after treatment. Improvement in firmness and hydration was reported by 22 patients (73.3%), whereas 7 patients (23.3%) reported no change and 1 patient (3.3%) reported worsening.

**Figure 4:**
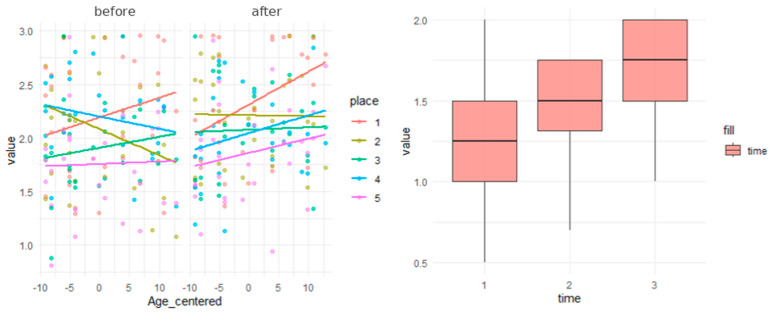
Change in puncture depth according to the treatment sequence.

Friedman’s test demonstrated a significant difference with *p* < 0.001 in the needle depth associated with pinpoint bleeding across subsequent treatment sessions. Nemenyi post hoc comparisons showed significantly greater needle depth during the second session compared with the first (*p* = 0.0036), during the third session compared with the first (*p* < 0.001), and during the third session compared with the second (*p* < 0.001). The median needle depth increased from 1.25 mm during the first session to 1.50 mm during the second and 1.75 mm during the third session (Figure 4).

## 4. Discussion

The present study showed heterogeneous, location-dependent variability in instrumental skin parameters after microneedling of the neck and décolleté, without evidence of a uniform global response. Statistically significant site-specific changes were observed mainly at point 4, although these findings should be interpreted cautiously given the exploratory design and limited sample size. Patient-reported improvement did not align with objective instrumental measurements, indicating a discrepancy between subjective and device-based outcomes.

Both corneometry and indentometry analyses indicated that anatomical location was the main factor influencing treatment response. Significant variability between measurement sites was observed both at baseline and after treatment, suggesting that regional differences in skin structure, thickness, photodamage, hydration, and mechanical properties may substantially affect the response to microneedling [9,21]. Interestingly, point 4 was the only location at which both increased hydration and improved firmness were demonstrated instrumentally, supporting the concept of spatial heterogeneity in skin remodeling after microneedling. However, a chance observation cannot be excluded given the small sample size, exploratory study design, and absence of a randomized comparative design. This site-specific finding, therefore, requires confirmation in larger cohorts. The absence of a generalized instrumental effect should be interpreted cautiously, particularly because patient-reported and instrumental outcomes assessed different aspects of treatment response. Corneometry demonstrated a statistically significant increase in skin hydration only at measurement point 4, with no significant changes at the remaining treated sites and no overall time effect in the model including the untreated control point. Similarly, indentometry showed a significant decrease in indentation values, corresponding to increased firmness, only at point 4, without a generalized improvement across the treated area. In contrast, 22 participants (73.3%) reported subjective improvement in both skin hydration and firmness after treatment. No significant associations were found between changes in corneometer or indentometer measurements and patient-reported assessments. Similar discrepancies between patient-reported outcomes and instrumental measurements have previously been described in esthetic medicine studies [12]. One possible explanation is that patient-reported outcomes may reflect aspects not captured by the selected instrumental methods; alternatively, microneedling alone may not have produced measurable changes within the study timeframe. Furthermore, subjective improvement may partly reflect placebo- or expectation-related effects, particularly in cosmetic procedures, where such responses are generally confined to patient perception rather than objective instrumental parameters.

Alternatively, the lack of generalized corneometer and indentometer changes may indicate that microneedling alone does not produce a meaningful improvement in hydration or firmness within the evaluated timeframe. Available studies frequently combine microneedling with adjunctive substances, which limits direct comparison with the present protocol [10,11,12,13]. The interpretation of indentometry results requires particular caution. Increased skin hydration may paradoxically increase susceptibility to indentation despite clinically perceived improvement in skin quality [21,24,25]. Consequently, lower stiffness measurements do not necessarily contradict subjective improvement and may partially reflect altered tissue hydration rather than worsening structural support.

Previous microneedling studies have also reported discrepancies between patient satisfaction and instrumental outcomes [9,11,12,13]. Microneedling-induced dermal remodeling is a gradual biological process involving collagen synthesis and extracellular matrix reorganization, with maximal effects potentially developing over several months [4,11,12,14,15,16]. In the present study, follow-up was limited to 4 weeks after the final treatment session, which may not have fully captured delayed remodeling effects. El-Domyati et al. demonstrated more pronounced histological changes after six treatment sessions [17], although their study evaluated facial skin only.

An additional observation was that the needle depth increased across subsequent sessions which was associated with pinpoint bleeding. Topical anesthesia was not used in the present study because of concerns that local anesthetics may interfere with cellular processes involved in tissue repair. However, the absence of anesthesia may have influenced both patient comfort and the achievable treatment depth. Although needle depth was individually adjusted according to the appearance of pinpoint bleeding, discomfort may have limited further penetration in some participants. Pain intensity and procedural comfort were not assessed using a standardized scale; therefore, their potential influence on treatment depth cannot be determined. Moreover, procedural protocols using topical anesthesia may permit different treatment depths and may not be directly comparable with the present findings. Consequently, the reported depth values and their progressive increase across sessions should be interpreted within the context of a non-anesthetized microneedling protocol.

An important strength of the present study is the use of objective bioengineering methods in an anatomical region that remains poorly represented in the literature. According to the PubMed search performed on 20 April 2026, only a very limited number of studies have evaluated microneedling outcomes using corneometry, whereas no studies using indentometry were identified. Moreover, publications specifically addressing microneedling of the neck and décolleté remain scarce. These observations highlight the limited evidence base and support the need for further prospective studies using standardized instrumental assessment methods.

The study also has several limitations. The relatively small sample size may have reduced statistical power, particularly in subgroup- and location-specific analyses. Although linear mixed models were used to account for repeated measurements within participants across time points and anatomical sites, the study may have been underpowered to detect smaller effects. Therefore, the findings should be interpreted as exploratory and require confirmation in larger, adequately powered studies. The lack of randomization and allocation concealment should be considered a limitation of the study. Although the within-subject design, with treated and untreated control points assessed in the same participant, was used to reduce interindividual variability in baseline skin parameters, the use of an adjacent untreated area may not fully exclude a potential local spillover effect. Therefore, conclusions about the absence of an overall time effect should be interpreted with caution and should be confirmed in randomized controlled studies. Furthermore, instrumental methods assess selected physical skin properties and may not fully reflect complex dermal remodeling processes occurring after microneedling. Future studies should include larger cohorts, longer follow-up periods, repeated measurements at each anatomical point, and standardized environmental conditions during instrumental assessment. The lack of standardized pain or comfort assessment limits interpretation of whether the absence of topical anesthesia influenced the achievable treatment depth.

## 5. Conclusions

Microneedling of the neck and décolleté was associated with heterogeneous, location-dependent changes in instrumental skin parameters, without evidence of a uniform global response. Patient-reported improvement did not align with instrumental measurements, possibly due to the short follow-up period or lack of objective improvements in skin hydration or firmness. The treatment depth increased across subsequent sessions. The results should be treated cautiously due to the exploratory nature of this study. Further prospective studies with standardized objective assessment, larger cohorts, randomized designs, and longer follow-up are needed to better characterize the effects of microneedling alone in the neck and décolleté region.

## Figures and Tables

**Table 1:** Summary of linear mixed-model analysis of corneometer readings before and after the treatment series.

Analysis Model	Effect	F Value	*p* Value	Interpretation
Including control point 0	Time	1.36	0.2526	No overall time effect
	Anatomical location	9.72	<0.001	Values differed between measurement sites
	Time × anatomical location	2.84	0.0178	Change over time differed by site
Excluding control point 0	Time	0.87	0.3590	No overall time effect
	Anatomical location	2.71	0.0337	Values differed between treated sites
	Time × anatomical location	3.36	0.0121	Change over time differed by treated site

**Table 2:** Age-adjusted linear mixed-model analysis of corneometer readings according to age, time, and anatomical location.

Effect	F Value	*p* Value	Interpretation
Age	0.08	0.775	No significant association with corneometry outcomes
Anatomical location	10.03	<0.001	Corneometer values differed between measurement sites
Time	1.37	0.252	No overall change after treatment
Age × anatomical location	1.94	0.091	No significant age-related difference between locations
Age × time	1.09	0.306	Age did not significantly affect change over time
Anatomical location × time	2.84	0.018	Change over time differed by measurement site

**Table 3:** Linear mixed-model analysis of indentometer readings according to time and anatomical location.

Effect	F Value	*p* Value	Interpretation
Time	0.53	0.4712	No significant overall change after treatment
Anatomical location	10.86	<0.001	Indentometer values differed between measurement sites
Time × anatomical location	2.4	0.0401	Change over time differed by measurement site

**Table 4:** Age-adjusted linear mixed-model analysis of indentometer readings according to age, time, and anatomical location.

Effect	F Value	*p* Value	Interpretation
Age	1.74	0.1981	No significant association with indentometry outcomes
Anatomical location	11.4	<0.001	Indentometer values differed between measurement sites
Time	0.56	0.4619	No overall change after treatment
Age × anatomical location	2.45	0.0369	Age-related associations differed by measurement site
Age × time	2.27	0.1427	Age did not significantly affect change over time
Anatomical location × time	2.4	0.0401	Change over time differed by measurement site

**Table 5:** Relationship between instrumental measurements and patient-reported assessments before and after treatment.

Instrumental Outcome	Effect	F Value	*p* Value	Interpretation
Corneometer	Patient-reported hydration	0.69	0.4103	No significant association
	Anatomical location	2.08	0.069	No significant location effect
	Time	4.72	0.0364	Significant overall time effect
	Patient-reported hydration × location	0.56	0.7288	No significant interaction
	Patient-reported hydration × time	3.87	0.0571	No significant interaction
	Location × time	2.18	0.0583	No significant interaction
Indentometer	Patient-reported firmness	0.16	0.692	No significant association
	Anatomical location	2.64	0.0237	Values differed between measurement sites
	Time	0.1	0.7586	No significant overall time effect
	Patient-reported firmness × location	1.11	0.3548	No significant interaction
	Patient-reported firmness × time	0.17	0.686	No significant interaction
	Location × time	2.22	0.0541	No significant interaction

## Data Availability

Dataset available on request from the authors.

## Appendix A

**Table A1:** Changes in corneometer readings after the treatment series at points 0–5 in the linear mixed model (LMM). Results of the Type III analysis of variance and model parameter estimates are presented.

Type III Analysis of Variance (Satterthwaite Method)						
**Factor**	**Sum Sq**	**Mean Sq**	**NumDF**	**DenDF**	**F**	***p*-Value**
time	236.1	236.07	1	29	1.3625	0.2526
place	8416.4	1683.28	5	145	9.7150	<0.001
time:place	2459.6	491.92	5	145	2.8391	0.0178
**Including Point 0**						
**Parameter**	**Estimate**		**Std. Error**	**df**	**t Value**	***p*-Value**
(Intercept)	33.413		3.138	195.329	10.646	<0.001
time_after	4.050		3.811	143.828	1.063	0.2897
place1	9.733		3.629	285.693	2.682	0.0077
place2	17.517		3.629	285.693	4.827	<0.001
place3	14.720		3.629	285.693	4.056	<0.001
place4	6.873		3.629	285.693	1.894	0.0592
place5	20.113		3.629	285.693	5.543	<0.001
time_after:place1	−1.846		4.806	145.000	−0.384	0.7015
time_after:place2	−7.627		4.806	145.000	−1.587	0.1148
time_after:place3	−2.823		4.806	145.000	−0.587	0.5579
time_after:place4	8.863		4.806	145.000	1.844	0.0672
time_after:place5	−5.363		4.806	145.000	−1.116	0.2663
**Excluding Point 0**						
**Factor**	**Sum Sq**	**Mean Sq**	**NumDF**	**DenDF**	**F**	***p*-Value**
time	156.5	156.50	1	29	0.8688	0.3590
place	1949.3	487.34	4	116	2.7055	0.0337
time:place	2420.9	605.23	4	116	3.3600	0.0121
**LMM Model Estimates (Excluding Point 0)**						
**Parameter**	**Estimate**	**Std. Error**	**df**		**t Value**	***p*-Value**
(Intercept)	43.1467	3.2295	172.0669		13.360	<0.001
time_after	2.2040	3.9556	119.2148		0.557	0.5785
place2	7.7833	3.6757	229.1674		2.118	0.0353
place3	4.9867	3.6757	229.1674		1.357	0.1762
place4	−2.8600	3.6757	229.1674		−0.778	0.4373
place5	10.3800	3.6757	229.1674		2.824	0.0052
time_after:place2	−5.7807	4.9007	115.9998		−1.180	0.2406
time_after:place3	−0.9773	4.9007	115.9998		−0.199	0.8423
time_after:place4	10.7093	4.9007	115.9998		2.185	0.0309
time_after:place5	−3.5173	4.9007	115.9998		−0.718	0.4743

**Table A2:** Corneometry—relationship between age, location, and time.

Type III Analysis of Variance (Satterthwaite Method)						
**Effect**	**Sum Sq**	**Mean Sq**	**NumDF**	**DenDF**	**F**	***p*-Value**
Age_centered	14.40	14.40	1	28	0.083	0.775
place	8689.50	1737.91	5	140	10.03	<0.001
time	236.78	236.78	1	28	1.37	0.252
Age_centered × place	1681.70	336.33	5	140	1.94	0.091
Age_centered × time	188.33	188.33	1	28	1.09	0.306
place × time	2459.60	491.92	5	145	2.84	0.018
**LMM Parameter Estimates for the Age-Adjusted Corneometry Model**						
**Parameter**	**Estimate**		**Std. Error**	**df**	**t Value**	***p*-Value**
(Intercept)	33.41333		3.13878	182.49815	10.645	<0.001
Age_centered	−0.06424		0.38400	112.69387	−0.167	0.8674
place1	9.73333		3.59662	280.64324	2.706	0.0072
place2	17.51667		3.59662	280.64324	4.870	<0.001
place3	14.72000		3.59662	280.64324	4.093	<0.001
place4	6.87333		3.59662	280.64324	1.911	0.0570
place5	20.11333		3.59662	280.64324	5.592	<0.001
time_after	4.05000		3.80947	141.18358	1.063	0.2895
Age_centered × place1	−0.66342		0.37657	139.99999	−1.762	0.0803
Age_centered × place2	−0.12617		0.37657	139.99999	−0.335	0.7381
Age_centered × place3	0.01687		0.37657	139.99999	0.045	0.9643
Age_centered × place4	0.45522		0.37657	139.99999	1.209	0.2288
Age_centered × place5	0.16821		0.37657	139.99999	0.447	0.6558
Age_centered × time_after	0.32431		0.31107	28.00001	1.043	0.3061
place1 × time_after	−1.84600		4.80648	144.99995	−0.384	0.7015
place2 × time_after	−7.62667		4.80648	144.99995	−1.587	0.1148
place3 × time_after	−2.82333		4.80648	144.99995	−0.587	0.5579
place4 × time_after	8.86333		4.80648	144.99995	1.844	0.0672
place5 × time_after	−5.36333		4.80648	144.99995	−1.116	0.2663

**Table A3:** Changes in indentometer readings after the treatment series at points 0–5 in the linear mixed model. Results of the Type III analysis of variance and model parameter estimates are presented.

Type III Analysis of Variance (Satterthwaite Method)						
**Factor**	**Sum Sq**	**Mean Sq**	**NumDF**	**DenDF**	**F**	***p*-Value**
time	0.0641	0.06411	1	29	0.5330	0.4712
place	6.5297	1.30594	5	145	10.8567	<0.001
time:place	1.4420	0.28840	5	145	2.3975	0.0401
**LMM Model Parameter Estimates (Including Point 0)**						
**Parameter**	**Estimate**		**Std. Error**	**df**	**t Value**	***p*-Value**
(Intercept)	2.3997		0.0827	262.3673	29.017	<0.001
time_after	−0.1153		0.1005	143.4020	−1.147	0.2532
place1	−0.2073		0.1034	272.8756	−2.004	0.0460
place2	−0.3197		0.1034	272.8756	−3.090	0.0022
place3	−0.4920		0.1034	272.8756	−4.756	<0.001
place4	−0.2020		0.1034	272.8756	−1.953	0.0519
place5	−0.6453		0.1034	272.8756	−6.239	<0.001
time_after:place1	0.2373		0.1266	145.0011	1.874	0.0629
time_after:place2	0.2453		0.1266	145.0011	1.937	0.0547
time_after:place3	0.2813		0.1266	145.0011	2.221	0.0279
time_after:place4	−0.0370		0.1266	145.0011	−0.292	0.7706
time_after:place5	0.2213		0.1266	145.0011	1.748	0.0826
**Analysis of Variance (Type III, Satterthwaite Method; Model Excluding Point 0)**						
**Factor**	**Sum Sq**	**Mean Sq**	**NumDF**	**DenDF**	**F**	***p*-Value**
time	0.1896	0.18958	1	29	1.4128	0.2442
place	4.2083	1.05208	4	116	7.8405	<0.001
time:place	0.9923	0.24808	4	116	1.8488	0.1242
**LMM Model Parameter Estimates (Excluding Point 0)**						
**Parameter**	**Estimate**		**Std. Error**	**df**	**t value**	***p*-Value**
(Intercept)	2.192333		0.086643	231.263136	25.303	<0.001
time_after	0.122000		0.105202	126.415191	1.160	0.2484
place2	−0.112333		0.109234	218.323451	−1.028	0.3049
place3	−0.284667		0.109234	218.323451	−2.606	0.0098
place4	0.005333		0.109234	218.323451	0.049	0.9611
place5	−0.438000		0.109234	218.323451	−4.010	<0.001
time_after:place2	0.008000		0.133758	115.999838	0.060	0.9524
time_after:place3	0.044000		0.133758	115.999838	0.329	0.7428
time_after:place4	−0.274333		0.133758	115.999838	−2.051	0.0425
time_after:place5	−0.016000		0.133758	115.999838	−0.120	0.9050

**Table A4:** Indentometry—relationship between age, location, and time (including point 0).

Type III Analysis of Variance (Satterthwaite Method)						
**Effect**	**Sum Sq**	**Mean Sq**	**NumDF**	**DenDF**	**F**	***p*-Value**
Age_centered	0.2090	0.20901	1	28	1.7376	0.1981
place	6.8555	1.37111	5	140	11.3984	<0.001
time	0.0669	0.06693	1	28	0.5564	0.4619
Age_centered × place	1.4715	0.29431	5	140	2.4467	0.0369
Age_centered × time	0.2736	0.27359	1	28	2.2744	0.1427
place × time	1.4420	0.28840	5	145	2.3975	0.0401
**LMM Parameter Estimates for the Age-Adjusted Indentometry Model**						
**Parameter**	**Estimate**		**Std. Error**	**df**	**t Value**	***p*-Value**
(Intercept)	2.399667		0.081440	254.918525	29.465	<0.001
Age_centered	0.008272		0.009913	160.581730	0.835	0.4052
place1	−0.207333		0.101890	268.846256	−2.035	0.0428
place2	−0.319667		0.101890	268.846256	−3.137	0.0019
place3	−0.492000		0.101890	268.846256	−4.829	<0.001
place4	−0.202000		0.101890	268.846256	−1.983	0.0484
place5	−0.645333		0.101890	268.846256	−6.334	<0.001
time_after	−0.115333		0.099816	143.379285	−1.155	0.2498
Age_centered × place1	0.010283		0.011234	140.000048	0.915	0.3616
Age_centered × place2	−0.026992		0.011234	140.000048	−2.403	0.0176
Age_centered × place3	−0.008108		0.011234	140.000048	−0.722	0.4716
Age_centered × place4	−0.011825		0.011234	140.000048	−1.053	0.2943
Age_centered × place5	−0.006543		0.011234	140.000048	−0.582	0.5612
Age_centered × time_after	0.012156		0.008060	27.999862	1.508	0.1427
place1 × time_after	0.237333		0.126644	144.999738	1.874	0.0630
place2 × time_after	0.245333		0.126644	144.999738	1.937	0.0547
place3 × time_after	0.281333		0.126644	144.999737	2.221	0.0279
place4 × time_after	−0.037000		0.126644	144.999737	−0.292	0.7706
place5 × time_after	0.221333		0.126644	144.999737	1.748	0.0826

**Table A5:** Type III analysis of variance (Satterthwaite method)—instrumental results and patients’ subjective assessments before and after the treatment series.

Corneometer Results						
**Effect**	**Sum Sq**	**Mean Sq**	**NumDF**	**DenDF**	**F**	***p*-Value**
NWL	119.88	119.88	1	55.970	0.6881	0.4103
place	1808.96	361.79	5	243.027	2.0766	0.0690
time	822.06	822.06	1	36.630	4.7185	0.0364
NWL × place	489.91	97.98	5	249.764	0.5624	0.7288
NWL × time	674.37	674.37	1	34.973	3.8708	0.0571
place × time	1896.60	379.32	5	193.065	2.1772	0.0583
**Indentometer Results**						
**Effect**	**Sum Sq**	**Mean Sq**	**NumDF**	**DenDF**	**F**	***p*-Value**
JDR	0.01908	0.01908	1	55.704	0.1585	0.6920
place	1.58827	0.31765	5	284.977	2.6393	0.0237
time	0.01150	0.01150	1	47.315	0.0955	0.7586
JDR × place	0.66842	0.13368	5	284.257	1.1107	0.3548
JDR × time	0.01991	0.01991	1	46.895	0.1655	0.6860
place × time	1.33660	0.26732	5	179.319	2.2211	0.0541
